# Analysis of Quality Distinctions of Pumpkin Seed Oil (*Cucurbita pepo* var. *oleifera*) and Walnut Oil (*Juglans regia* L.)

**DOI:** 10.3390/molecules31081263

**Published:** 2026-04-11

**Authors:** Kamil Czwartkowski, Edyta Nizio, Damian Marcinkowski, Dominik Kmiecik, Anna Grygier, Aleksander Siger, Wojciech Golimowski

**Affiliations:** 1 Department of Agroengineering and Quality Analysis, Faculty of Production Engineering, Wroclaw University of Economics and Business, Komandorska 118/120, 53-345 Wroclaw, Poland; edyta.nizio@ue.wroc.pl (E.N.); wojciech.golimowski@ue.wroc.pl (W.G.); 2 Department of Inorganic Chemistry, Faculty of Production Engineering, Wroclaw University of Economics and Business, Komandorska 118/120, 53-345 Wroclaw, Poland; damian.marcinkowski@ue.wroc.pl; 3 Department of Food Technology of Plant Origin, Faculty of Food Science and Nutrition, Poznan University of Life Sciences, Wojska Polskiego 31, 60-634 Poznan, Poland; dominik.kmiecik@up.poznan.pl (D.K.); anna.grygier@up.poznan.pl (A.G.); 4 Department of Food Biochemistry and Analysis, Faculty of Food Science and Nutrition, Poznan University of Life Sciences, Wojska Polskiego 31, 60-634 Poznan, Poland; aleksander.siger@up.poznan.pl

**Keywords:** pumpkin seed oil, walnut oil, niche oils, edible oil composition

## Abstract

The study aimed to characterize the quality and evaluate the content of bioactive substances in cold-pressed pumpkin seed and walnut oils obtained from the specific varieties (*Cucurbita pepo* var. *oleifera* and *Juglans regia* L.). The analyses included the determination of acid value, peroxide value, and anisidine value. The content of chlorophylls and carotenoids was identified, and the fatty acid, phytosterol, and tocopherol profiles were characterized. The results were subjected to principal component analysis and compared with the physicochemical parameters of other popular niche oils. It was shown that both oils tested have unique, relatively simple fatty acid profiles (only 5–6 dominant acids were identified). In addition, significant differences in squalene content were observed: pumpkin seed oil showed a higher concentration than other vegetable oils. In contrast, walnut oil was found to lack squalene, which is atypical among the analyzed niche oils.

## 1. Introduction

In recent years, there has been an increase in interest in so-called niche oils, i.e., oils produced on small-scale equipment from seeds and fruits [1]. The search for original sensory experiences and health benefits is focused on the processing of unconventional species, such as berries, cereals, legumes, and species with biotechnological potential. They are a source of bioactive compounds, such as phytosterols, tocopherols, carotenoids, phospholipids, and polyphenols [2].

The process of obtaining them is not standardized, which results in significant differences in the composition and properties of oils, depending on the species, variety, growing conditions, seed moisture content, and the technology used to obtain them (cold and hot pressing, supercritical CO_2_ extraction, or microwave-assisted extraction) [3]. Many studies indicate that the oil extraction method significantly affects the oxidative stability, fatty acid profile, and antioxidant activity of the final product [4].

Scientific literature also emphasizes the frequent synergy among bioactive compounds in niche oils, which endows them with unique health-promoting properties, such as anti-inflammatory, cardioprotective, and neuroprotective effects [5]. In addition, depending on the degree of refinement, these oils may contain both nutrients (e.g., essential fatty acids and fat-soluble vitamins) and anti-nutrients (e.g., glycosides, tannins, and phytic acid) [6]. Therefore, one of the important areas of contemporary research is the optimization of oil purification processes to preserve their natural biological potential while ensuring product safety and stability.

The extraction of oil from pumpkin seeds (*Cucurbita* spp.) is a process with a long tradition, especially in Central Europe (Austria, Hungary, Slovenia, southern Poland). It has high culinary and health values—it contains significant amounts of unsaturated fatty acids (UFAs), mainly linoleic acid (C18:2, known as n-6) and oleic acid (C18:1, known as n-9), as well as phytosterols, phenolic compounds, and vitamins from the E group, including α- and γ-tocopherol [7]. These compounds have strong antioxidant properties, protecting cell membrane lipids from peroxidation, which is important in preventing cardiovascular disease [8].

The raw material in the form of seeds can be obtained from any pumpkin variety, e.g., *Cucurbita maxima* or *Cucurbita moschata*. However, the most desirable are the shell-less varieties of *Cucurbita pepo* var. *oleifera*, due to their lack of a hard shell and their high seed fat content (up to 50%). In recent years, genetic and agronomic research has also been conducted to improve oil pumpkin varieties, including increasing γ-tocopherol content and oil oxidative stability [9,10]. Before pressing, pumpkin seeds are roasted (at 100–120 °C). The process of roasting seeds before pressing serves three purposes: it imparts characteristic aroma and color, increases pressing efficiency, and deactivates lipolytic enzymes. This process also involves Maillard reactions, which occur when amino acids in proteins react with reducing sugars at high temperatures. This results in the formation of hundreds of organic compounds (melanoidins) with flavoring and coloring properties. In addition, chlorophyll degradation occurs, leading to the formation of compounds that can both enrich the sensory profile (e.g., by degrading polyphenols) and reduce nutritional value (e.g., by degrading tocopherols) [11,12]. For this reason, contemporary technological research focuses on developing gentle thermal processing methods (e.g., microwave drying, ultrasonic processing) that minimize the loss of bioactive components. Another interesting area of research is the use of press residues—pumpkin seed meal—as a source of functional protein, dietary fiber, and bioactive peptides with antioxidant and antihypertensive potential [13].

Walnut oil (*Juglans regia* L.) is obtained from walnut kernels, which contain up to 72% fat. It is characterized by a high content of polyunsaturated fatty acids (PUFA) among all vegetable oils—especially linoleic acid (n-6) and α-linolenic acid (n-3)—which gives it high nutritional value but also low oxidative stability [14,15]. Contemporary research on walnut oil focuses, among other things, on improving its shelf life by using natural antioxidants, such as extracts from rosemary, green tea, or sage, as well as microencapsulation techniques (e.g., maltodextrin or whey proteins) [16,17].

From a biochemical point of view, walnut oil is a valuable source of phytosterols (approx. 200–250 mg/100 g) and tocopherols (approx. 40 mg/100 g), with γ-tocopherol being the dominant form [18,19]. Recent studies suggest that consumption of this oil may have a beneficial effect on the blood lipid profile, vascular endothelial function, and cognitive function, thanks to its phenolic compounds and PUFAs with neuroprotective properties [20]. Due to this oil’s susceptibility to oxidation, an important research challenge is to develop storage conditions and innovative packaging methods (e.g., protective atmospheres, dark glass, biodegradable packaging with oxidation inhibitors) that extend its shelf life without the need for chemical stabilization [21,22].

As shown in previous studies [23], both pumpkin seed oil and walnut oil have attracted considerable consumer interest on the Polish market. In the context of the dynamic development of the functional food market, it is necessary to determine the quality characteristics and biochemical markers of authenticity of these oils, including through fatty acid profiling using GC-FID, determination of tocopherol content using HPLC, and phytosterol content using GC, as well as spectral analysis (NIR, FTIR) to identify adulteration.

Therefore, this study aims to characterize the quality and bioactive properties of niche oils—pumpkin seed oil and walnut oil—obtained from the most used raw materials, with respect to their health potential.

## 2. Results and Discussion

### 2.1. Organoleptic Characteristics of the Oils Obtained

**Figure 1:**
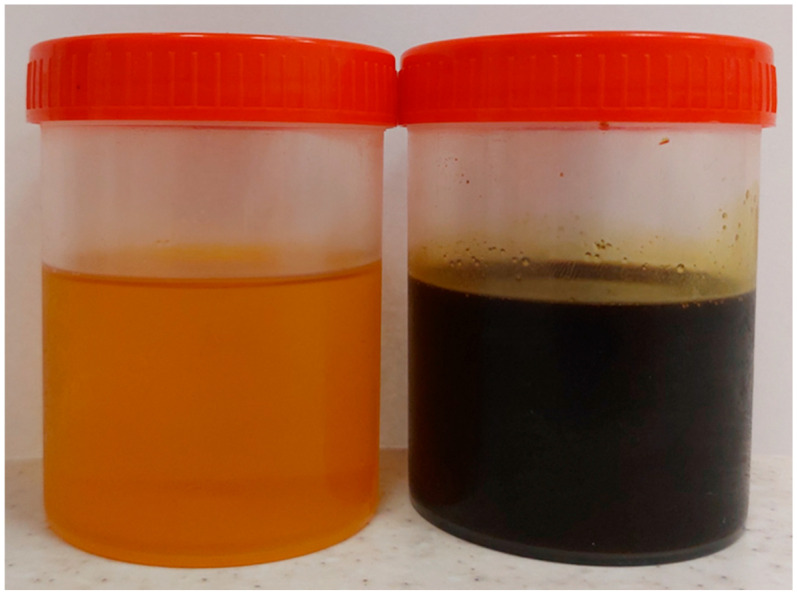
Walnut oil (**left**) and pumpkin seed oil (**right**).

The pumpkin oil had an intense dark green-burgundy color. The oil was clear, with a small amount of sediment at the bottom of the container. The aroma was very intense and characteristic of roasted pumpkin. No foreign aromas were detected. The taste was slightly nutty, moderately intense, typical of roasted pumpkin oil. The walnut oil, on the other hand, was light brown in color and highly transparent. There was no sediment at the bottom of the container. The aroma was not very intense, with a noticeable nutty scent and no foreign aromas. The taste was not very distinctive, with a slightly noticeable aftertaste of the raw material. Both oils are shown in Figure 1.

### 2.2. Discussion of Instrumental Analysis

The parameters of pumpkin seed oil (PSO*) and walnut oil (WO*) obtained were compared with those of popular niche oils reported in the literature. The comparison was based on data for: pumpkin seed oil (PSO), walnut oil (WO), olive oil (OO), rapeseed oil (RO), sesame oil (SO), linseed oil (FO), soybean oil (SBO), palm oil (PO), grape seed oil (GO), peanut oil (AO), hemp oil (HO), and corn oil (CO). The results are presented in Table 1, Table 2, Table 3, Table 4, Table 5 and Table 6.

Compared to the analyzed vegetable oils, PSO* is distinguished by its unusual color, as indicated by a comparison of oil colors in the CIE Lab color space (Table 1). Referring to PSO obtained by Ordoñez Lozada et al. (2021), the color of PSO* is lighter, as indicated by a*, which means a more intense red color of the oil, most likely due to the presence of melanoidins formed as a result of the Maillard reaction during the roasting of the raw material before pressing [24]. The color of WO* in terms of photometric brightness (L*) is most similar to OO, RO, and differs significantly from WO analyzed by Fediuc et al. (2025), who obtained walnut oil with a highly dark color [25]. There are also significant differences in the b* parameter between WO* and WO. The oil we obtained had a more intense yellow color. The differences in walnut oil color are most likely due to the pre-treatment of the raw material.

**Table 2:** Comparison of oil fatty numbers (AV [mg KOH/g of oil], POV [meq O_2_/kg of oil], AnV and TOTOX [-]).

	AV	POV	AnV	TOTOX	References
PSO*	1.23 ± 0.04	5.43 ± 0.37	4.89 ± 0.30	15.73 ± 0.52	[-]
WO*	1.19 ± 0.04	4.58 ± 0.07	2.04 ± 0.17	10.17 ± 0.15	[-]
PSO	0.14–0.29	0.98–16.40	3.61–4.53	5.57–37.33	[24]
WO	0.05–1.52	0.94–4.14	0.47–1.09	2.35–9.37	[38,39,40,41]
OO	0.20–4.20	5.00–17.40	2.60–8.10	12.60–42.90	[42]
RO	0.17–1.37	0.45–1.65	nd–1.96	0.90–5.26	[43,44]
SO	0.63–7.51	1.10–18.20	1.91–7.53	4.11–43.93	[45,46,47,48]
FO	0.60–0.90	0.75–1.28	0.45–1.14	1.95–3.70	[49]
SBO	0.05–2.09	0.79–1.99	1.58–4.68	3.16–8.66	[50,51]
PO	0.42–1.95	0.52–3.26	0.65–2.02	1.69–8.54	[52,53,54]
GO	0.26–2.20	4.88–7.33	7.24–8.73	17.00–23.39	[48,55,56]
AO	0.46–1.41	1.58–12.90	0.75–4.68	3.91–30.48	[57,58]
HO	0.70–1.76	1.94–22.40	0.11–3.58	3.99–48.38	[59,60,61,62]
MO	0.11–4.01	0.49–3.31	8.08–9.32	9.06–15.94	[63,64]

nd—not detected.

As shown in Table 2, PSO* and WO* obtained meet the standards of Codex Alimentarius (CODEX-Stan 210:1999), which specify a maximum AV of 4.0 mg KOH/g of oil and a POV of 15 meq O_2_/kg of oil [65]. In the literature, higher AVs correspond to oil obtained at high temperatures, as was the case, for example, in the work of Willenberg et al. (2019) for olive oil [42]. In turn, higher POVs are most often the result of poor-quality raw material with high moisture content, as indicated by studies by Chau et al. (2021) and Liang et al. (2018) [47,59]. Another parameter that determines the degree of oil degradation is AnV, which has no recommended values but affects TOTOX. AnV values should be as low as possible. Both PSO* and WO* have AnV values below the literature average for crude oils.

**Table 3:** Comparison of natural dye content [µg/g].

	Chlorophylls	Carotenoids	Σ	References
PSO*	71.77 ± 8.66	25.61 ± 2.74	97.38 ± 23.01	[-]
WO*	7.05 ± 0.10	9.69 ± 0.13	16.73 ± 1.32	[-]
PSO	46.00–159.00	8.21–27.40	54.21–186.40	[24]
WO	0.13–3.97	14.22–15.44	14.35–19.41	[40,66]
OO	0.15–61.96	0.53–31.51	0.68–93.47	[6,67]
RO	4.32–44.49	52.60–358.70	56.92–403.19	[6,68]
SO	nd-0.89	0.74–6.83	0.74–7.82	[69,70]
FO	1.14–130.50	2.75–76.90	3.89–207.40	[6,71]
SBO	0.13–158.76	12.31–122.53	12.44–281.39	[72]
PO	nd-4.36	30.00–988.00	30.00–992.36	[73,74,75]
GO	1.00–9.11	2.60–598.50	3.60–607.11	[76]
AO	nd-0.72	0.18–1.80	0.18–2.52	[6,57,58]
HO	56.30–84.00	23.40–53.00	79.70–137.00	[59,77]
MO	nd-4.90	nd-307.50	nd-312.40	[6,78,79]

nd—not detected.

Chlorophylls have pro-oxidative properties, while carotenoids have antioxidant properties. Their content depends on the raw material from which the oil is derived [71]. WO* is characterized by a very low total content of natural pigments (approx. 16.73 µg/g), which is within the literature average for WO, according to Elouafy et al. (2022) and Górnaś et al. (2014) [40,66]. In turn, PSO* contains more than five times more natural pigments than WO*, which may result in better oxidative stability of this oil (Table 3). Referring to the content of these pigments in the study by Ordoñez Lozada et al. (2021), PSO* has an average content in pumpkin oils [24]. In addition, PSO* is characterized by a high content of natural pigments, in particular chlorophylls, compared to other oils analyzed.

Table 4 shows the profiles of essential fatty acids in raw niche oils. Compared to other oils, PSO* and WO* are characterized by a very short fatty acid profile (PSO* consists of only 6 fatty acids and WO* of 5 fatty acids). A short fatty acid profile may contribute to slower fat oxidation. The fatty acid profile of walnut oil is included in Codex Alimentarius (CODEX Stan 210:1999) [65]. However, pumpkin seed oil is not included in this list of standards. Therefore, the fatty acid profile analysis was based on the study by Ordoñez Lozada et al. (2021), who reported similar fatty acid profiles in pumpkin oils [24].

**Table 4:** Comparison of fatty acid profiles (FAPs) [%].

	PSO*	WO*	PSO	WO	OO	RO	SO	FO	SBO	PO	GO	AO	HO	MO
C16:0	11.68 ± 0.04	6.63 ± 0.00	13.2–16.4	6.0–8.0	7.5–20.0	1.5–6.0	7.9–12.0	4.0–11.3	8.0–13.5	39.3–47.5	5.5–11.0	5.0–14.0	6.2–6.6	8.6–16.5
C18:0	5.90 ± 0.11	1.38 ± 0.01	11.2–15.8	1.0–3.0	0.5–5.0	0.5–3.1	4.5–6.7	2.0–8.0	2.0–5.4	3.5–6.0	3.0–6.5	1.0–4.5	3.1–3.6	nd-3.3
C18:1	31.30 ± 0.11	14.48 ± 0.05	28.7–40.7	14.0–23.0	55.0–83.0	8.0–60.0	34.4–45.5	9.8–36.0	17.0–30.0	36.0–44.0	12.0–28.0	35.0–80.0	11.3–12.4	20.0–42.2
C18:2	50.49 ± 0.04	61.25 ± 0.03	26.7–29.0	54.0–65.0	3.5–21.0	11.0–23.0	36.9–47.9	8.3–30.0	48.0–59.0	9.0–12.0	58.0–78.0	4.0–43.0	54.0–55.0	34.0–65.6
C18:3	0.21 ± 0.00	12.25 ± 0.02	0.6–1.9	9.0–15.4	0.0–1.0	5.0–13.0	0.2–1.0	43.8–70.0	4.5–11.0	nd-0.5	nd-1.0	nd-0.5	20.1–21.6	nd-2.0
C20:0	0.42 ± 0.00	nd	1.1–1.9	nd-0.3	0.0–0.6	nd-3.0	0.3–0.7	nd-1.0	0.1–0.6	nd-1.0	nd-1.0	0.7–2.0	0.3–1.3	0.3–1.0
C20:1	nd	nd	nd	nd-0.3	0.0–0.4	3.0–15.0	nd-0.3	nd-1.2	nd-0.5	nd-0.4	nd-0.3	0.7–3.2	0.4–0.6	0.2–0.6
C20:2	nd	nd	nd	nd	nd	nd-1.0	nd	nd	nd-0.1	nd	nd	nd	0.0–0.1	nd-0.1
C22:0	nd	nd	nd	nd-0.2	0.0–0.2	nd-2.0	nd-1.1	nd-0.5	nd-0.7	nd-0.2	nd-0.5	1.5–4.5	0.3–0.6	nd-0.5
C22:1	nd	nd	nd	nd	nd	2.0–60.0	nd	nd-1.2	nd-0.3	nd	nd-0.3	nd-0.6	nd	nd-0.3
C22:2	nd	nd	nd	nd	nd	nd-2.0	nd	nd	nd	nd	nd	nd	nd	nd
C24:0	nd	nd	nd	nd	0.0–0.2	nd-2.0	nd-0.3	nd-0.3	nd-0.5	nd	nd-0.4	0.5–2.5	nd-0.3	nd-0.5
C24:1	nd	nd	nd	nd	nd	nd-3.0	nd	nd	nd	nd	nd	nd-0.3	nd	nd
References	[-]	[-]	[24]	[65]	[80,81,82]	[65]	[65]	[65]	[65]	[65]	[65]	[65]	[35]	[65]

nd—not detected.

**Table 5:** Comparison of phytosterol profiles (PSPs) and squalene content.

[%]	PSO*	WO*	PSO	WO	OO	RO	SO	FO	SBO	PO	GO	AO	HO	MO
Cholesterol	nd	1.5 ± 0.0	nd	nd	nd	nd-1.3	0.1–0.5	nd	0.2–1.4	2.6–7.0	nd-0.5	nd-3.8	nd	0.2–0.6
Brassicasterol	nd	nd	nd	nd	nd	5.0–13.0	0.1–0.2	nd-1.0	nd-0.3	nd	nd-0.2	nd-0.2	nd	nd-0.2
Campesterol	nd	8.1 ± 0.2	0.8–2.0	4.0–6.5	1.2–9.0	24.7–38.6	10.1–20.0	25.0–31.0	15.8–24.2	18.7–27.5	7.5–14.0	12.0–19.8	15.0–15.8	16.0–24.1
Stigmasterol	nd	nd	nd	nd	0.9–7.3	0.2–1.0	3.4–12.0	7.0–9.0	14.9–19.1	8.5–13.9	7.5–12.0	5.4–13.2	1.6–2.3	4.3–8.0
β-sitosterol	5.8 ± 0.3	63.0 ± 1.1	7.1–7.6	70.0–92.0	36.4–52.8	45.1–57.9	57.7–61.9	45.0–53.0	47.0–60.0	50.2–62.1	64.0–70.0	47.4–69.0	61.4–66.8	54.8–66.6
Δ-5-avenasterol	nd	5.2 ± 0.2	nd	0.5–6.0	nd-10.3	2.5–6.6	6.2–7.8	8.0–12.0	1.5–3.7	nd-2.8	1.0–3.5	5.0–18.8	7.3–7.6	1.5–8.2
Δ-7-stigmasterol	50.4 ± 2.5	nd	nd	nd-3.0	nd	nd-1.3	0.5–7.6	nd	1.4–5.2	0.2–2.4	0.5–3.5	nd-5.1	nd	0.2–4.2
Δ-7-avenasterol	18.2 ± 1.1	nd	40.7–43.9	nd-2.0	nd	nd-0.8	1.2–5.6	nd	1.0–4.6	nd-5.1	0.5–1.5	nd-5.5	2.7–4.5	0.3–2.7
Others	25.6 ± 2.0	22.2 ± 1.6	46.5–51.5	nd	20.6–26.1	nd-4.2	0.7–9.2	nd	nd-1.8	nd	nd-5.1	nd-1.4	5.4–9.5	nd-2.4
Σ PS [mg/100 g]	450.0 ± 15.6	135.0 ± 4.7	54.5–62.7	50–176	211.7–288.0	450–1130	450–1900	230–690	180–450	30–70	200–700	90–290	195–213	700–2210
Squalene [mg/g]	2.10 ± 0.10	nd	0.59–3.35	0.09–0.12	1.5–7.5	0.02–0.13	0.09–0.61	0.02–0.83	0.02–0.92	0.07–0.48	0.12–0.13	0.05–1.33	nd	0.01–2.57
References	[-]	[-]	[6,7]	[6,65]	[6,80,83]	[6,65]	[6,65]	[6,65]	[6,65]	[6,65]	[6,65]	[6,65]	[35]	[6,65]

nd—not detected.

As shown in Table 5, PSO* is characterized by a high content of phytosterols (approximately 450 mg/100 g) and squalene (approximately 2.10 mg/g). In this respect, PSO* is a distinctive niche oil. Higher values have indeed been reported in the literature for other oils; e.g., flaxseed oil has a phytosterol content of up to 690 mg/100 g, according to the Codex Alimentarius (CODEX-Stan 210:1999) [65]. However, this is uncommon, as indicated by the research of Tian et al. (2023) [6]. No squalene was detected in WO*, which is also unusual, as only HO is characterized by the absence of squalene, according to earlier research by Golimowski et al. (2022), which compared the sterol profiles of hemp oils obtained from three varieties of hemp at two temperature variants [35].

**Table 6:** Comparison of tocopherol content (TC) [mg/kg].

	α-T	β-T	γ-T	δ-T	References
PSO*	103.2 ± 0.8	1.2 ± 0.0	452.9 ± 3.0	6.3 ± 0.1	[-]
WO*	12.5 ± 0.2	1.1 ± 0.1	283.9 ± 1.1	28.9 ± 0.3	[-]
PSO	7.4–88.5	nd	54.1–397.3	10.4–45.0	[7,24]
WO	nd-170.0	nd-110.0	120.0–400.0	nd-60.0	[65]
OO	264.3–290.0	2.55–10.0	10.0–15.90	1.0–4.46	[6,83]
RO	100.0–386.0	nd-140.0	189.0–753.0	nd-22.0	[65]
SO	nd-3.3	nd	521.0–983.0	4.0–21.0	[65]
FO	2.0–265.0	nd	100.0–712.0	nd-14.0	[65]
SBO	9.0–352.0	nd-36.0	89.0–2307.0	154.0–932.0	[65]
PO	4.0–193.0	nd-234.0	nd-526.0	nd-123.0	[65]
GO	16.0–38.0	nd-89.0	nd-73.0	nd-4.0	[65]
AO	49.0–373.0	nd-41.0	88.0–389.0	nd-22.0	[65]
HO	2.7–53.0	nd-5.8	594.0–967.0	3.2–50.3	[84,85]
MO	23.0–573.0	nd-356.0	268.0–2468.0	23.0–75.0	[65]

nd—not detected.

In the case of tocopherols (Table 6), PSO* has a significantly more diverse profile than the pumpkin oils reported in the literature. In addition, it stands out in this respect from other niche oils, particularly in terms of its α- and γ-tocopherol content. On this basis, it can be concluded that PSO* is an excellent source of vitamin E. WO* contains significantly less tocopherols, but their content is within the literature average. Both oils obtained exhibit a very low β-tocopherol content, which prolongs their oxidative stability [83]. However, this is compensated for by other tocopherols.

**Figure 2:**
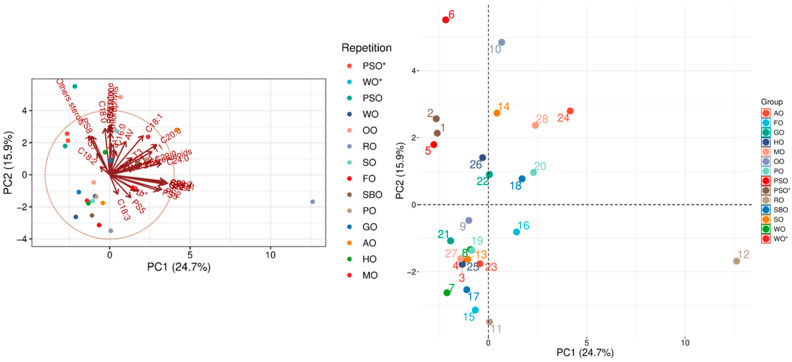
PCA for the analyzed oils.

Analysis of the principal components of the physicochemical parameters of the analyzed oils effectively grouped the oils. The first two principal components explained 40.6% of the total variance, with PC1 accounting for 24.7% and PC2 for 15.9%, indicating reduced dimensionality and greater component separation. PC1 serves as the main compositional gradient, reflecting the inverse relationship among the analyzed quality indicators (especially the fatty acid profile, sterol profile, and tocopherol content). PC2, on the other hand, takes into account changes in color and the associated change in natural pigment content, as well as the analyzed fatty acid values (AV, POV, AnV). Analysis of Figure 2 suggests that PSO* is significantly different from other oils, but falls within the ranges specified in the literature for pumpkin seed oil (PSO). On the other hand, WO* does not fall within the ranges defined in the literature for walnut oil (WO). PCA clusters this oil based on the analyzed parameters, placing it close to variants of corn oil (MO), palm oil (PO), sesame oil (SO), peanut oil (PO), and hemp oil (HO).

### 2.3. Additional Parameters Analyzed

In addition to the physicochemical parameters of the oils analyzed, compared, and statistically evaluated, other parameters of PSO* and WO* were also determined, namely water content (WC), phosphorus content (P), and oxidative stability (OS). WO* was characterized by a significantly higher water content (approximately 920.10 ± 27.77 mg H_2_O/kg) than PSO* (390.60 ± 9.79 mg H_2_O/kg). In the study by Golimowski et al. (2022), the water content of oils obtained from three hemp varieties was determined to range from 121 to 159 mg H_2_O/kg [35]. Therefore, it can be concluded that PSO* and WO* exhibit a high water content. The water content in oils results mainly from the condition of the raw material from which they are obtained. In turn, PSO* was characterized by more than ten times higher phosphorus content (P = 422.00 ± 5.35 mg/kg) than WO* (38.40 ± 3.12 mg/kg). In the literature, phosphorus content is correlated with phospholipids, which are mainly oil contaminants. The significantly higher P content in pumpkin seed oil is not only due to differences in the raw material but also to heat treatment of the raw material before pressing [86]. For example, in the study by Łaska-Zieja et al. (2020), the phosphorus content in rapeseed oil was determined to be 95–150 mg/kg [87]. It can be concluded that PSO* contains significantly more phospholipid impurities than WO*, which results in a much lighter color of WO*.

PSO* would likely have a significantly longer oxidative stability than WO* (Table 7). These results are consistent with previous reports of antioxidant components, such as natural pigments and tocopherols, which are present in significantly greater amounts in PSO* than in WO*. A limitation of this method is that the oxidation process is accelerated in an oxygen atmosphere and under increased pressure, so degradation mechanisms may differ in ambient conditions. It should also be noted that under normal storage conditions, factors such as light exposure, potential interactions with the packaging, and the presence of other gases in the air come into play. Furthermore, the induction time depends primarily on the established process conditions, namely temperature, pressure, and sample volume. It is possible to compare the results obtained under the same oxidation conditions.

## 3. Materials and Methods

### 3.1. Oil Pressing

#### 3.1.1. Pumpkin Seed Oil

Pumpkin seeds (*Cucurbita pepo* var. *oleifera*, Gleisdorfer Ölkürbis variety), with an oil content of 45%, were imported from Austria, where they were grown. Before pressing, the pumpkin seeds were roasted. First, they were heated for 15 min at 100–105 °C, then for another 5 min at 110–115 °C, another 5 min at 115–117 °C, and finally for the last 5 min at 117–120 °C. The raw material was then cooled to 20 °C. The seeds were placed in a twin-screw press and pressed at a temperature not exceeding 40 °C. After pressing, the oil was stored at 6 °C for 3 days to allow solid impurities to settle. The oil was then decanted. The sample prepared in this way was subjected to laboratory analysis.

#### 3.1.2. Walnut Oil

Walnuts (*Juglans regia* L., Franquette variety), with a kernel oil content of 68%, were imported from France, where they were grown. Before pressing, the nuts were shelled and then heated to 20 °C. The prepared raw material was placed in a twin-screw press and pressed at a temperature not exceeding 40 °C. After pressing, the oil underwent a procedure similar to that used for pumpkin seed oil.

### 3.2. Color Using the CIE Lab Method

The color in the CIE Lab space was determined using a HunterLab Vista device (HunterLab Europe GmbH, Murnau, Germany). Three milliliters of the sample were measured and placed in a quartz spectrophotometric cuvette with an optical path length of 10 mm. The measurement was performed in total transmission mode (TTRAN) with the device calibrated to a blank for an empty quartz cuvette.

### 3.3. Acid Value (AV)

AV was determined in accordance with PN-EN ISO 660 [88]. In an Erlenmeyer flask, approximately 2 g of the analyzed oil was weighed and then dissolved in a diethyl ether/ethanol mixture (1:1 *v*/*v*). The weighed portion of walnut oil was dissolved in 25 cm^3^ of the solvent mixture. Pumpkin seed oil, which exhibited a dark green color, was dissolved in 250 cm^3^ of the diethyl ether/ethanol mixture. The obtained mixture was titrated with 0.01 M potassium hydroxide solution in the presence of an alcoholic phenolphthalein solution. The titration continued until the indicator turned pink (raspberry shade). Each oil sample was titrated three times. The acid value was expressed in mg KOH/g of oil and calculated using the following formula:

$$AV=\;\frac{C_{KOH}\times (V-V_{0})\times M_{KOH}}{m}$$

where

*C_KOH_*—concentration of KOH solution [mol·dm^−3^],
*V*—volume of standard KOH solution used to titrate the analyzed oil sample [cm^3^],
*V*_0_—volume of standard KOH solution consumed to titrate the blank sample [cm^3^],
*M_KOH_*—molar mass of KOH [g·mol^−1^],
*m*—mass of analyzed sample [g].

### 3.4. Peroxide Value (POV)

POV was determined according to PN-EN ISO 3960 [89]. In an Erlenmeyer flask, a sample of the analyzed oil weighing 2 g (with an accuracy of 1 mg) was weighed. The walnut oil sample was then dissolved in 10 cm^3^ of chloroform. To this prepared mixture, 1 cm^3^ of freshly prepared saturated KI solution and 15 cm^3^ of glacial acetic acid were added, and the flask was tightly capped with a stopper and shaken for 1 min. Next, the flask was set aside in a dark place for 5 min. After this time, 75 cm^3^ of distilled water and 5 drops of 5% potato starch solution were added to the flask, turning the solution dark blue. The sample prepared in this way was titrated with a standard sodium thiosulfate solution at 0.01 mol·dm^−3^. The endpoint of titration is the decolorization of the solution, which persists for at least 30 s. The pumpkin seed oil samples, due to their dark green color, were dissolved in a tenfold greater amount of solvent. The quantities of the remaining reagents remained unchanged.

The value of the peroxide number of the analyzed oil sample, expressed in milliequivalents of active oxygen per kg of oil [meq O_2_/kg of oil], was calculated using the following formula:

$$POV=\frac{\left(1000\times C\times (V-V_{0}\right)}{m}$$

where

*C*—concentration of Na_2_S_2_O_3_ solution 0.01 mol·dm^−3^,
*V*—volume of standard Na_2_S_2_O_3_ solution used to titrate the analyzed oil sample [cm^3^],
*V*_0_—volume of standard Na_2_S_2_O_3_ solution consumed to titrate the blank sample [cm^3^],
*m*—mass of analyzed sample [g].

### 3.5. Anisidine Value (AnV)

AnV was determined according to PN-EN ISO 6885:2016 [90]. At the beginning, the anisidine reagent was prepared by dissolving 0.125 g of p-anisidine in glacial acetic acid in a 50 cm^3^ flask. In a 25 cm^3^ volumetric flask, 3.1 g of the analyzed oil sample was weighed and then dissolved in iso-octane to a volume of 25 cm^3^. Subsequently, 5 cm^3^ of the sample was transferred into two test tubes; 1 cm^3^ of the anisidine reagent was added to one of them, and 1 mL of glacial acetic acid to the other. For the third test tube, 5 cm^3^ of iso-octane and 1 cm^3^ of the anisidine reagent were taken (blank sample). The test tubes were sealed with stoppers and thoroughly mixed. They were then left in a dark place at room temperature for 8 min. After this time, the absorbance of all three samples was measured in quartz cuvettes against iso-octane.

The anisidine value was calculated using the following formula:

AnV=100×Q×V ×m [1.2×(A1−A2−A0)]

where

*Q*—content of the sample in the measured solution, based on which the anisidine value is expressed [g∙cm^−3^], Q = 0.01 g∙cm^−3^,
*V*—volume in which the test sample was dissolved [cm^3^], V = 25 cm^3^,
*m*—mass of analyzed sample [g],
1.2—correction factor resulting from the dilution of the test solution with 1 cm^3^ of the anisidine reagent or glacial acetic acid,
*A*0—absorbance of the unreacted test solution (oil and glacial acetic acid),
*A*1—absorbance of the reacted test solution (oil and anisidine reagent),
*A*2—absorbance of the blank sample (iso-octane and anisidine reagent).

### 3.6. TOTOX

TOTOX (Total Oxidation Value) index was calculated by formula [91]:

$$TOTOX=\left(2\;\times POV\right)+AnV$$

### 3.7. Natural Dyes

Chlorophyll and carotenoid content were determined spectrophotometrically according to the method proposed by Alan Wellburn [92]. One milliliter of pumpkin seed oil was collected and weighed into a glass test tube, then 20 cm^3^ of methanol or 2 cm^3^ of walnut oil were added. The test tube was sealed with a stopper and shaken until the oil was entirely suspended in the solvent. The sample was then centrifuged using a mini vortex mixer and left in a dark room for 15 min to separate the alcohol phase from the fat phase. After this time, 3 cm^3^ of the alcohol phase was taken and filtered through a 0.45 µm hydrophobic PTFE syringe filter to obtain a clear supernatant. It was then placed in a quartz spectrophotometric cuvette with an optical path length of 10 mm. The absorbance of the clear supernatant was measured at three wavelengths: λ = 665.2 nm, λ = 652.4 nm, and λ = 470.0 nm using a SHIMADZU UV-1800 spectrophotometer (SHIMADZU Corporation, Kyoto, Japan). The analysis was performed in three replicates. The content of chlorophyll a (C_a_), chlorophyll b (C_b_), and carotenoids (C_x_) was determined from the following formulas:

$$C_{a}=\frac{\left(16.72\;A_{665.2}-9.16\;A_{652.4}\right)\times D}{m}$$

$$C_{b}=\frac{\left(34.09\;A_{652.4}-15.28\;A_{665.2}\right)\times D}{m}$$

$$C_{x}=\frac{\left(\frac{1000\;A_{470.0}-1.63\;C_{a}-104.96\;C_{b}\;}{221}\right)\times D}{m}$$

where

*D*—amount of solvent added [cm^3^],
*m*—weight of oil collected [g].

### 3.8. Fatty Acid Profile (FAP)

Gas chromatography was used to determine the fatty acid profile by measuring fatty acid methyl esters. The oils were saponified with 0.5 M KOH in methanol. Transesterification of fatty acids with BF3 (boron trifluoride) solution in methanol was performed according to the official method of the American Oil Chemists’ Society (AOCS) Ce 2–66 [93]. A 7890 A series gas chromatograph (Agilent Tech. Inc., Santa Clara, CA, USA) with an injection volume of 1.0 µL and a split ratio of 1/50. A J & W Scientific HP-88 series fused silica capillary column with dimensions of 100 m × 0.25 mm × 0.20 μm (Agilent Tech. Inc., Santa Clara, CA, USA) and a flame ionization detector (FID) from Agilent Tech. Hydrogen was used as the carrier gas at a head pressure of 1.5 mL/min. The supplement gas flows (air, hydrogen, and helium) through the FID (Agilent Tech. Inc., Santa Clara, CA, USA) were 450, 40, and 30 mL/min, respectively. The detector and injector temperatures were set at 280 °C and 250 °C, respectively. The initial column temperature of 120 °C was held for 1 min, then increased to 175 °C at 10 °C/min and maintained for 10 min. It was then increased to 210 °C at 5 °C/min, held for 5 min, and then increased to 230 °C at 5 °C/min, held for 5 min. The FAME were identified by comparing with those of a commercial Supelco 37 Component FAME Mix (Merck KGaA, Darmstadt, Germany). All reagents used were of GC or HPLC purity. The results are expressed as percentages (%) of total fatty acids.

### 3.9. Phytosterol Profile (PSP)

The phytosterol content was determined in three replicates using gas chromatography with a Hewlett-Packard 6890 chromatograph (Agilent Technologies, Palo Alto, CA, USA) in splitless mode with an FID and a DB-35MS capillary column (25 m × 0.20 mm, 0.33 μm; Agilent J&W, Folsom, CA, USA). A 0.05 g sample of oil was taken, and 50 µg of the internal standard (5α-cholestane, Supelco, Bellefonte, PA, USA) was added. The samples were saponified with 1 M KOH in methanol, and the unsaponifiable substances were extracted with a hexane:methyl tert-butyl ether (1:1, *v*/*v*) mixture. The solvent was evaporated under nitrogen, and the dry residue was dissolved in anhydrous pyridine (Supelco, Bellefonte, PA, USA) and silylated with BSTFA + 1% TMCS (Supelco, Bellefonte, PA, USA). The detector and injector were set to 300 °C. The oven temperature was initially set to 100 °C for 5 min, then increased at 25 °C/min to 250 °C, and then at 3 °C/min to 290 °C. The final temperature was maintained for 20 min. The carrier gas was hydrogen, and the flow rate was 1.5 mL/min. The phytosterol content was determined by comparing their retention times with those of the standards. Results were expressed in mg/100 g and converted to % content to generate a plant sterol profile.

### 3.10. Tocopherol Content (TC)

Oil samples were dissolved in n-hexane and injected into a Waters HPLC system (Waters, Milford, MA, USA) equipped with a fluorometric detector (Waters 474) and a photodiode-array detector (Waters 2998 PDA). The separation of tocopherols was performed using a LiChrosorb Si 60 column (250 × 4.6 mm, 5 μm, Merck, Darmstadt, Germany), thermostated at 20 °C using a Jetstream 2 Plus column oven. The mobile phase consisted of a mixture of n-hexane and 1,4-dioxane (96:4, *v*/*v*), with a flow rate of 1.0 mL/min. The injection volume was 10 μL. The excitation wavelength was set to λ = 295 nm, and the emission wavelength to λ = 330 nm. Quantitative analysis was performed using a 2475 fluorometric detector (Waters, Milford, MA, USA), while a photodiode array detector was used as an additional tool for the identification of tocochromanols based on UV-Vis spectra. The analyses were performed twice, and the results were expressed in mg/kg of oil. Tocochromanol standards with a purity of over 95% were purchased from Calbiochem–Merck4Biosciences (Darmstadt, Germany).

### 3.11. Phosphorus Content

A 50 g sample of oil was calcined at 650 °C in platinum evaporators prior to analysis, and the residue was dissolved in hydrochloric acid HCl (1 + 5, *v*/*v*). The tests were performed using a simultaneous emission spectrometer iCAP 7400+ MFC Duo (Thermo Fisher Scientific Inc., Waltham, MA, USA), which was used for the tests, utilizing an Echelle optics system with a CID semiconductor array detector, characterized by an extended spectral range from 166 nm to 847 nm and high resolution, with a dual plasma observation system: radial and axial. The instrument enables the determination of trace levels of a broad spectrum of elements in various matrices (detection limits for most elements at the ppb level).

### 3.12. Water Content

The water content was determined by Karl Fischer coulometric titration using an Aquamax KF Coulometric titrator (GR Scientific Ltd., Ampthill, UK). 1.00 g ± 0.05 g of the tested oil was added to the anodic solution. The measurement was performed three times. The device reports the water content of the sample as mg H_2_O/kg of oil.

### 3.13. Oxidative Stability

The oxidative stability of the oils was determined using the RapidOxy 100 device (Anton Paar GmbH, Graz, Austria) at 80, 100, 120, and 140 °C. It was decided to perform the analysis at four different temperatures, as the device can estimate oxidation time under various temperature conditions based on this analysis. Using an automatic pipette, 5 mL of the sample was placed in the device’s heating chamber, which was filled with technical oxygen at 700 kPa. The sample is then heated to the set temperature, which is maintained throughout the measurement. The pressure inside the chamber increases to a maximum (<1000 kPa). During this time, wear causes the oil to oxidize, leading to a drop in chamber pressure. A pressure drop of 10% of the maximum pressure is considered to be the moment of complete oxidation of the sample. The oxidative stability of the oil is determined as the oxidation induction time, which shows how long the sample resists decomposition under accelerated conditions.

### 3.14. Statistical Analysis

Statistical analysis was performed using the SRplot platform developed by Tang et al. (2023) [94]. To assess the possibility of grouping oils according to their physicochemical parameters, principal component analysis (PCA) was performed. The mean ranges of data from the literature were analyzed and compared with triplicate laboratory analyses. The results were arranged in a summary matrix, and Bartlett’s test was performed in Statistica 13.3 (StatSoft, Krakow, Poland). The test demonstrated statistical significance of the correlation matrix (*p* < 0.05), confirming the existence of a relationship between the variables. Based on this, the data were deemed suitable for principal components analysis. Values not detected were omitted from the analysis.

## 4. Conclusions

The analyses provide detailed information on the characteristic properties of pumpkin seed oil and walnut oil, compared with other vegetable oils, such as rapeseed and sunflower oils. It should be emphasized that a limitation of the comparison is the varied pretreatment of the oilseed material in the analyzed studies. Therefore, the analysis based on the literature data should be supplemented in the future with empirical studies that will allow for the unambiguous identification of the factors determining the quality of these oils. Both oils have a relatively simple fatty acid profile, typically comprising 5–6 dominant compounds. Walnut oil is dominated by linoleic acid (C18:2, n-6), whose content may exceed 50–60%, with a significant proportion of α-linolenic acid (C18:3, n-3) at around 10–15%, resulting in a favorable n-6 to n-3 fatty acid ratio compared to many commonly used vegetable oils. Pumpkin seed oil, on the other hand, contains high amounts of linoleic acid (approx. 45–60%) and oleic acid (approx. 20–40%), with a low content of α-linolenic acid. A significant difference between the oils under analysis is their squalene content. Pumpkin seed oil contains relatively high amounts of this compound (in the range of several hundred mg/kg).

In contrast, in walnut oil, it is present at trace levels or sometimes undetectable. Pumpkin seed oil is also distinguished by a higher content of natural antioxidants, including tocopherols (especially γ-tocopherol), carotenoids, and chlorophylls, which translates into greater oxidative stability, as evidenced by a longer induction period and a slower increase in the peroxide value during storage. In contrast, walnut oil, due to its high content of polyunsaturated fatty acids, is more susceptible to oxidation processes. Both oils also possess characteristic plant sterol profiles (including β-sitosterol, campesterol, and stigmasterol) and specific tocopherol compositions, which can serve as markers of the raw material’s authenticity. Pumpkin seed oil is distinguished by its intense color, ranging from dark green to brown, resulting from the presence of chlorophylls and phosphorus compounds, including phospholipids.

Walnut oil shows greater water permeability during pressing from the raw material, which may affect quality parameters such as acid value and polar compound content. Based on their chemical composition, both oils can be considered valuable dietary components, providing essential unsaturated fatty acids, phytosterols, and natural antioxidants with potential health benefits. Further research should include integrated sensory analyses to assess the organoleptic acceptability of the refined oils, as well as analyses of volatile compound composition, which will provide a better understanding of the impact of the adsorbents used.

## Figures and Tables

**Table 1:** Comparison of oil colors in the CIE Lab color space.

	L*	a*	b*	References
PSO*	1.75 ± 0.01	12.59 ± 0.01	2.99 ± 0.02	[-]
WO*	92.44 ± 0.00	−1.75 ± 0.01	52.20 ± 0.00	[-]
PSO	5.12–11.65	1.93–5.07	−1.08–0.77	[24]
WO	20.55–21.13	−1.28–(−0.70)	4.70–5.74	[25]
OO	90.31–95.80	25.49–42.23	94.39–95.78	[26]
RO	90.35–95.50	2.77–5.48	87.00–88.60	[27,28]
SO	96.60–98.00	0.70–1.50	13.40–16.00	[27]
FO	60.05–63.71	3.28–6.10	91.08–99.80	[29,30]
SBO	69.35–78.05	−12.43–(−10.07)	82.79–84.71	[31]
PO	54.07–80.37	21.73–33.49	37.58–82.45	[32]
GO	30.52–47.61	2.78–4.10	5.65–26.85	[33]
AO	24.02–25.09	−0.86–(−0.67)	34.53–35.41	[34]
HO	22.03–23.59	1.12–2.04	2.84–5.47	[35]
MO	33.45–40.20	−1.80–5.12	9.89–10.70	[36,37]

**Table 7:** Oxidative stability at different temperatures.

	Temperature [°C]	Induction Time [hh:mm:ss]
PSO*	140	00:42:33
	120	02:36:31
	100	11:10:50
	80	60:50:26
WO*	140	00:20:29
	120	00:51:56
	100	03:12:14
	80	18:12:04

## Data Availability

The original contributions presented in this study are included in the article. Further inquiries can be directed to the corresponding author.

## Abbreviations

The following abbreviations are used in this manuscript:

UFA	unsaturated fatty acid
PUFA	polyunsaturated fatty acids
GC-FID	gas chromatography—flame ionization detector
HPLC	high-performance liquid chromatography
NIR	near-infrared spectroscopy
FTIR	Fourier transform infrared spectroscopy
PSO*	obtained pumpkin seed oil
WO*	obtained walnut oil
PSO	pumpkin seed oil reported in the literature
WO	walnut oil reported in the literature
OO	olive oil reported in the literature
RO	rapeseed oil reported in the literature
SO	sesame oil reported in the literature
FO	flaxseed oil reported in the literature
PO	palm oil reported in the literature
GO	grape seed oil reported in the literature
AO	peanut oil reported in the literature
HO	hemp oil reported in the literature
CO	corn oil reported in the literature
AV	acid value
POV	peroxide value
AnV	anisidine value
TOTOX	Total Oxidation Value
α-T	α-tocopherol
β-T	β-tocopherol
γ-T	γ-tocopherol
δ-T	δ-tocopherol
PCA	principal component analysis
WC	water content
P	phosphorus content
OS	oxidative stability
TTRAN	total transmission mode
PTFE	poli(tetrafluoroetylen)
BSTFA	N,O-Bis(trimethylsilyl)trifluoroacetamide
TMCS	trimetylochlorosilan
